# Medical students’ experience of personal loss: incidence and implications

**DOI:** 10.1186/1472-6920-13-36

**Published:** 2013-03-06

**Authors:** Rebecca Whyte, Thelma Quince, John Benson, Diana Wood, Stephen Barclay

**Affiliations:** 1 General Practice and Primary Care Research Unit, Department of Public Health and Primary Care, University of Cambridge, Institute of Public Health, Robinson Way, Cambridge, UK; 2 School of Clinical Medicine, University of Cambridge, Clinical Medical School, Cambridge Biomedical Campus, Hills Road, Cambridge, UK

**Keywords:** Undergraduate education, Bereavement, Student wellbeing, Pastoral care

## Abstract

**Background:**

Medical students are generally young people, often away from home for the first time and undertaking a course in which they are learning to care for people at all stages of life, including those approaching death. Existing research indicates that their experiences of personal bereavement may have significant implications for their pastoral welfare and medical learning. No previous studies have tracked medical student experience of bereavement longitudinally and no recent data are available from the UK.

**Aims:**

The study aims to identify medical students’ experience of personal bereavement: the prevalence prior to and during the course and their relationship with those who died.

**Method:**

Paper and online questionnaire including questions about recent personal loss. Setting / Participants: Four cohorts of core science and clinical medical students at the University of Cambridge, 1021 participants in total.

**Results:**

Mean response rate was 65.2% for core science students and 72.8% for clinical students. On entry to the core science course, 23.1% of all students had experienced a loss at some point. Between 13.0% and 22.5% experienced bereavement during years 1 – 5 of the course: some (1.3% - 6.3%) experienced multiple or repeated losses. Close deaths reported were most commonly those of grandparents followed by friends.

**Conclusions:**

Medical students commonly experience close personal bereavement, both before and during their course. Educators need to be aware of the range of personal and educational implications of bereavement for medical students, and ensure that appropriate help is available. Further research could explore incidence of loss at other medical schools and investigate the impact and depth of experience of loss.

## Background

Bereavement is a stressful life event with short and long-term effects on physical and mental health [1-6]. Between 35 - 48% of university students are reported to have experienced bereavement in the previous 24 months [3], at a time when they are away from family and other support networks and may not seek help [3]. Lower academic achievement has been reported in the term during which a bereavement occurs [3].

Bereavement has particular implications for medical students. Their course involves mastering a large volume of material delivered at a fast pace and learning to care for dying patients is an important part of their curriculum. It is estimated that in their first year after qualification, junior doctors on average care for 40 patients who die and many more with advanced disease [7,8]. Some medical students and junior doctors have negative views of end of life care: many report feeling stressed and unprepared when dealing with dying patients and their relatives [9-13]. Difficulties with dying patients may be intensified if the patient reminds them of someone close who has died [9,12,14,15].

It is therefore important to understand the extent to which medical students encounter personal bereavement. Studies have found around 15% of medical students have experienced the death of a family member in the previous year [15-17]. Most research has focused either on losses during the past year or whole life experience: some studies specify which bereavements should be regarded as significant (for example family members or, less frequently, family members and close friends) [15-18]. None have tracked medical student experience of bereavement longitudinally and no recent data are available from the UK.

The School of Clinical Medicine at the University of Cambridge is undertaking a longitudinal cohort study of the study of factors in medical student education which influence the quality of patient care in subsequent medical practice [19,20]. Data from this study concerning one such factor, student experience of personal bereavement, is reported in this paper.

### Aims

To investigate medical students’ experience of personal bereavement with regard to:

1] The prevalence prior to commencing the course

2] The prevalence during the course

3] Their relationship with those who had died

## Methods

The medical course at Cambridge comprises a Core Science component (Years 1–3) and a Clinical component (Years 4–6). Two hundred and eighty students, typically aged 18, enter the Core Science course. At the end of Year 3, half of these continue into the Clinical component; the others transfer to London and other medical schools for their clinical studies. From September 2007, all students entering Years 1 and 4 (the first years of the Core Science and Clinical components respectively) were invited to take part in a longitudinal study comprising an annual questionnaire survey. This contained validated instruments concerning anxiety, depression, death anxiety and student attitudes towards end life care and questions concerning personal experience of bereavement. This paper relates to participating students entering the Core Science and Clinical components in 2007–2010.

Two questions focused on personal experience of recent loss of someone close.

For new participants: “Have you experienced the death of someone close to you recently? If ‘yes’, please tell us who that was and when they died, using the table below”. For students repeating their participation: “Have you experienced the death of someone close to you in the last 12 months? If ‘yes’, please tell us who that was and when they died, using the table below”. No definitions of “recently” or “someone close to you” were given: this was left to participants to define. Reports of deaths occurring several years ago were interpreted as being of continuing significance.

Questionnaires were sent out early in each new academic year: all were paper-based in 2007 and 2008 and in 2009 for clinical component students, with an online version in 2009 for Core Science component students and for all students in 2010. Questionnaires were labelled by study number only and a data manager (who had no access to results) sent one reminder after 2 weeks. Participation was voluntary, with a prize awarded annually to a small number of participants. A summary of the data was circulated to staff involved in student pastoral care in the medical school and across the wider University. The study was approved by the University of Cambridge Psychology Ethics Committee.

Data were analysed using SPSS 18. Since only half of the Core Science component students remain in Cambridge for their Clinical training, separate analyses were undertaken for these two components. To assess the incidence of personal bereavement prior to starting the course (aim 1), analysis included all Year 1 students in 2007 – 2010. Students who did not respond on entry but did so in subsequent years 2 or 3 were also examined in order to identify bereavements which had occurred prior to the start of their course: their reported losses which occurred before the start of the Core Science course were backfitted and included alongside the Year 1 responses. The same backfitting process was repeated for all students who responded in Years 4 to 6: as their responses were several years after the start of the Core Science course and the questionnaire asked about “recent losses”, it is likely that these data underestimate losses.

To examine the incidence of personal bereavement during the course (aim 2), analysis included all students entering the Core Science 2007–2010 and the Clinical components 2007–2010 who answered in each year of the course. Responses in each year of the course were used to give information about losses in the previous 12 months, producing a year-by-year account of losses during the course.

A logistic regression analysis was undertaken to determine whether experience of loss before the start of either course component (Years 1 and 4), or experience of loss during either course component, affects study participation in subsequent years. Outcome variables were full participation at Years 2 and 3 for students entering Year 1 and full participation at Years 5 and 6 for students entering year 4. The explanatory variables were experience of loss before the course and experience of loss during the course. Year of course entry was included as an explanatory factor variable to adjust for student cohort. To investigate repeated and multiple losses, analysis included students entering the Core Science and Clinical components in 2007, 2008 and 2009 who maintained their participation for all years of their respective course so providing data relating to before the course component, and bereavements occurring during Years 1 and 2 or Years 4 and 5.

To invest igate the relationship with those who had died, (aim 3), analysis included all data for aims 1 and 2, coded according to relationship to the deceased.

## Results

Mean response rate was 65.2% for core science students and 72.8% for clinical students. Response rates varied between the cohorts, ranging from 54.8% to 82.2% (Table 1). In the completed cohorts 1 – 3, 224 Core Science (42.0% of total participants) and 157 Clinical students (53.2% of total participants) maintained their participation in all three years. (Cohort 4 will complete participation in 2012/2013). Mean age of respondents was 18.5 years for Year 1 and 21.4 years for Year 4. Respondents were representative of their years in terms of gender: 472 (46.3%) were male and 546 (53.6%) female. Gender was the only variable available for non-responders. Results of logistic regression analysis showed that experiencing a loss before the start of either the Core Science or Clinical components did not predict subsequent non-response (data not shown).

**Table 1:** Numbers of students participating and point in course when participated for the first time

	**Core science component**				
	**Total number of entrants**	**Total number of participants *(% of entrants*)**	**Number participating for the first time**		
			**Year 1**	**Year 2**	**Year 3**
Core Science Cohort 1: Students entering 2007	266	202 *(75.9%)*	183	13	6
Core Science Cohort 2: Students entering 2008	283	155 *(54.8%)*	140	13	2
Core Science Cohort 3: Students entering 2009	281	175 *(62.3%)*	155	9	11
Core Science Cohort 4: Students entering 2010	282	193 *(68.4%)*	189	4	n/a
**Total**	**1112**	**725 *(65.2%)***	**667**	**39**	**19**
	Clinical component*				
	**Total number of entrants**	**Total number of participants *(% of entrants)***	**Number participating for the first time**		
			**Year 4**	**Year 5**	**Year 6**
Clinical Cohort 1: Students entering 2007	135	111 *(82.2%)*	104	3	4
Clinical Cohort 2: Students entering 2008	135	104 *(77.0%)*	101	2	1
Clinical Cohort 3: Students entering 2009	135	80 *(59.3%)*	72	2	6
**Total**	**405**	**295 *(72.8%)***	**277**	**7**	**11**

*Students entering the Clinical component in 2010 were those who had entered the Core Science component in 2007.

Table 2 shows experience of loss prior to starting the two course components and overall. Amongst Core Science students 668 answered the questionnaire on entry at Year 1: a further 57 answered the questionnaire for the first time in subsequent years. 28.5% of core science respondents experienced a loss at some point before starting medical school: of these 15.3% experienced a loss in the 12 months prior to starting medical school and 13.2% experienced a more distant loss.

**Table 2:** Experience of loss before the course


**Students reporting losses prior to starting to study medicine**				
		**Numbers of students reporting loss prior to start of course**		
		**Loss in previous 12 months**	**Loss >12 months previously**	**Total**
Participants entering core science component 2007-2010				
Participants answering Year 1	n =668	108	94	202
Participants answering first time in Years 2 & 3	n =57	3	2	5
All participants	n = 725	111 (15.3%)	96 (13.2%)	207 (28.5%)
Participants entering clinical component 2007-2009				
All participants	n = 295	22 (7.5%)	7 (2.4%)	29 (9.8%)
Participants entering core science component 2007–2010 and clinical component 2007-2009				
All participants	n =1020	133 (13.0%)	103 (10.1%)	236 (23.1%)

Table 3 shows experience of loss during the course. These figures were derived from the question relating to losses experienced during the previous 12 months, hence the information provided by 432 students who responded in Year 2 related to losses they had experienced as Year 1 students.

**Table 3:** Number of students experiencing losses during the previous year of the course

**Year of course**	**Year 2**	**Year 3**	**Year 4**	**Year 5**	**Year 6**
Participants	432	390	345	262	182
Students experiencing loss	69 (16.0%)	53 (13.3%)	45 (13.0%)	54 (20.6%)	41 (22.5%)

Examination of the extent to which students experienced repeated losses ( i.e. one death in more than one year) and / or multiple losses (more than one death in any one year) considered only those students who maintained their participation for all three years of their course component: 224 Core Science and 157 Clinical students. During Years 1 and 2 of the Core Science component 24 (10.7%) students reported a loss both prior to the start of the course and during the course and a further 9 reported repeated losses during the course. Three students (1.3%) reported multiple losses (two (0.8%) during Year 1 and 1 (0.4%) during Year 2). During years 4 and 5 of the Clinical component 19 (12.1%) students reported loss of someone close both before and during the course and a further 9 (5.7%) reported repeated losses during the course. Ten students (6.3%) reported multiple losses. Of these 10 reported two losses in one year (5 (3.2%) in each of Years 4 and 5), and 1 (0.6%) reported three (Table 4).

**Table 4:** Experience of multiple and repeated losses during first two years of each course component

	**Repeated losses**		**Multiple losses**
	**Losses both prior to start of course and during the course**	**Repeated losses during the course**	
	Core Science Component		
Students maintaining participation for all 3 years of component n = 224	24 (10.7% )	9 (4.0%)	3 (1.3%)
	Clinical component		
Students maintaining participation for all 3 years of component n = 157	19 (12.1%)	9 (5.7%)	10 (6.3%)

Close deaths reported were most commonly those of grandparents, followed by friends. Deaths of siblings and parents were least common (Table 5).

**Table 5:** Relationship with those who died (%) of all deaths

	**Core science component**	
	**On entry to course**	**During course**
Grandparent	169 (64.5%)	37 (48.7%)
Friend	36 (13.7%)	14 (18.4%)
Parent or sibling	17 (6.5% )	2 (2.6%)
Other relative or friend	40 (15.3%)	23 (30.2%)
Total number of deceased	262	76
	**Clinical component**	
	**On entry to course**	**During course**
Grandparent	65 (55.6%)	34 (44.2%)
Friend	18 (15,4%)	18 (23.4%)
Parent or sibling	12 (10.3%)	1 (1.3%)
Other relative or friend	22 (18.8%)	24 (31.2%)
Total number of deceased	117	77

## Discussion

Experience of close personal loss was common amongst medical students both before and during the course. On entry to the Core Science course, around one quarter of respondents reported that they had experienced a recent close bereavement, nearly half of these losses having occurred in the preceding 12 months. This figure is likely to underestimate losses before the course. Between 13.0% and 22.5% of students experienced bereavement during each year of the course: some (1.3% - 6.3%) experienced multiple or repeated losses. The deaths reported were of people considered close: the largest category was grandparents (44.2% - 64.5%) as might be expected given the students’ ages. Deaths of friends were frequently reported (13.7% - 23.4%): although not all were age-group peers, the impact of losses of these bereavements might be very profound. The majority of students who experienced bereavement had only had one loss, although a significant minority had experienced combinations of multiple and / or repeated losses. This diversity, and differences in the personal relationship of those who had died, implies a wide range of student experience.

Figures from our study are broadly similar to the 26% of Scottish junior doctors who reported the loss of close family member or friend in the previous year [16,18,21]. They are lower than reports of American university student populations [1] and higher than the 14.8% and 16% found in studies of US medical schools [15,16].

The literature suggests that the implications of these data for the pastoral care and academic progress of students are wide-ranging. Experience of bereavement is particularly pertinent for medical students, for whom recent loss can affect reactions to gross anatomy teaching and dissection during basic science training [22-24]. During clinical training, relationships with patients may be affected: students with more experience of loss are more likely to avoid visiting and communicating with dying patients and their family members and find patient death more difficult where it reminds them of their relatives [9,14,25]. Students who have lost a parent or sibling in childhood may be less likely to want a close family member informed of a fatal disease diagnosis than those who have not suffered loss [26]. Recent experience of loss can make instruction about death and dying particularly difficult: when death of a family member occurs during a death education course, students are at risk of developing persistent anxiety, avoiding situations involving death or dying, and being less able to retain course content [25]. Bereavement may therefore be seen as a negative influence which needs to be taken into account when designing medical curricula in general and end of life care education courses in particular.

A countervailing view in the literature is that personal bereavement, if processed well, can be a positive experience for students both personally and professionally. For some, the experience is a positive factor in their motivation to study medicine [4]. A lack of experience of loss can leave medical students unprepared: the newness of experience of patient death can create strong emotional responses, leaving them unable to approach patient death in a “calm, rational and supportive” way [9,27]. Personal bereavement may thus be a positive learning experience, creating greater ability to overcome stereotypical approaches to patient needs. Those with experience of loss may be more realistic about what dying patients might experience and have a stronger belief in patients’ abilities to prepare for and accept death than their fellow students [28]. Doctors and medical students use previous personal experience to inform their professional understanding of how to care for people during bereavement; those with personal or professional experience of death have more positive attitudes towards and better knowledge about end of life care [7,12,29-31]. Other studies have suggested that experience of personal bereavement or illness could usefully be more explicitly harnessed and put to use in clinical practice [12,32].

Understanding the prevalence of personal bereavement among students is a first step towards implementing a strategy that ensures appropriate support is offered. The authors have disseminated a summary of these data to staff involved in student pastoral care within the medical school and across the wider University to raise awareness of these issues.

These data also have important implications for medical students’ future patient care as qualified doctors, since junior (and senior) doctors frequently care for patients approaching the end of life [7,8,18]. Despite significant efforts in the UK and other countries to increase the amount of palliative care education in the medical curricula of all medical schools [33-36], junior doctors still find communicating with dying patients and their relatives difficult and stressful [9] and feel unsupported. Skilled and sensitive support during periods of emotional stresses such as bereavement enables medical students to address and process their experiences in a fashion that will then enable them to provide care for dying patients in the future. Medical schools could usefully develop cultures in which students are encouraged to address personal issues in a mature way, avoiding a culture of “the mask of relaxed brilliance” that sees such an approach as sign of weakness [37].

Limitations of this study are acknowledged. The study was conducted at one medical school with high academic entry criteria and a strong emphasis on core science in the first three years: this might limit generalizability to other medical student populations. Response rates were variable and there was attrition longitudinally. Missing value analysis supports the generalizability of results to Cambridge medical students. While the structured questionnaire obtained large numbers of responses concerning the prevalence and nature of losses, qualitative information concerning the impact or depth of these experiences is lacking. The present study was unable to investigate the implications of bereavement for students’ academic achievement during the course or the ways in which they coped with loss and sought support. These important issues could usefully be addressed in a future study. The authors are currently establishing a repeat study at other UK medical schools.

Nevertheless, this study’s description of medical student experience of personal loss over several years’ student intake, and a longitudinal component is unique. This description of experience of bereavement before and during medical school raises important questions for pastoral care and curriculum design that could usefully be addressed in all medical schools.

## Conclusions

Medical students commonly experience close personal bereavement, both before and during their course. This experience will affect individual students in disparate ways, with a range of impacts on their learning. Educators and pastoral care staff need to be aware of the high prevalence of loss, the diversity of student experience and the range of personal and educational implications; and then ensure that appropriate help and support is available. Students could also be encouraged to reflect on their experience of loss and how it has affected their attitudes. It is important that tutors and students address these issues to ensure that the doctors of tomorrow are adequately prepared for their work in caring for dying patients.

## Consent

This study was approved by the University of Cambridge Psychology Ethics Committee, which gives approval for research studies involving students. The questionnaire started with the following: "I have read and understood the attached information sheet and agree to participate in this study". Students were asked NOT to sign the questionnaire as their responses were confidential. No patient consent was obtained as no patients were involved and no patient information was reported.

## Competing interests

The authors declare they have no competing interests.

## Authors’ contribution

The study was designed by TQ, JB, DW and SB. TQ led data collection, supported by James Brimicombe. RW, TQ and SB led data analysis and all authors contributed to the paper. All authors read and approved the final manuscript.

## Pre-publication history

The pre-publication history for this paper can be accessed here:

http://www.biomedcentral.com/1472-6920/13/36/prepub
